# A Method for Bridging Population-Specific Genotypes to Detect Gene Modules Associated with Alzheimer’s Disease

**DOI:** 10.3390/cells11142219

**Published:** 2022-07-16

**Authors:** Yulin Dai, Peilin Jia, Zhongming Zhao, Assaf Gottlieb

**Affiliations:** Center for Precision Health, School of Biomedical Informatics, University of Texas Health Science Center at Houston, Houston, TX 77030, USA; yulin.dai@uth.tmc.edu (Y.D.); peilin.jia@uth.tmc.edu (P.J.); zhongming.zhao@uth.tmc.edu (Z.Z.)

**Keywords:** dense gene modules, gene expression imputation, genetically regulated expression (GReX), Alzheimer’s disease

## Abstract

Background: Genome-wide association studies have successfully identified variants associated with multiple conditions. However, generalizing discoveries across diverse populations remains challenging due to large variations in genetic composition. Methods that perform gene expression imputation have attempted to address the transferability of gene discoveries across populations, but with limited success. Methods: Here, we introduce a pipeline that combines gene expression imputation with gene module discovery, including a dense gene module search and a gene set variation analysis, to address the transferability issue. Our method feeds association probabilities of imputed gene expression with a selected phenotype into tissue-specific gene-module discovery over protein interaction networks to create higher-level gene modules. Results: We demonstrate our method’s utility in three case-control studies of Alzheimer’s disease (AD) for three different race/ethnic populations (Whites, African descent and Hispanics). We discovered 182 AD-associated genes from gene modules shared between these populations, highlighting new gene modules associated with AD. Conclusions: Our innovative framework has the potential to identify robust discoveries across populations based on gene modules, as demonstrated in AD.

## 1. Introduction

The use of genome-wide association studies (GWAS) to detect associations between genotyped data and specific traits has seen a large growth in the recent decade, owing to improved technology and decreasing costs of DNA sequencing. Transcriptome imputation techniques [1,2] further extend the GWAS methodology by using genotype information to predict transcription levels of genes and later using these imputed transcription levels in a transcription-wide association study to boost the power to detect novel diseases [3,4,5,6]. However, it is now widely acknowledged that broadening the diversity of studied populations will improve the effectiveness of GWAS [7] with an especially noticeable difference between African and non-African populations [8]. Correspondingly, substantial variations between populations are also observed in transcriptome imputation methods [9] like PrediXcan [1].

Here, we assume that gene modules might be more robust across populations than individual genes. The strengths of this approach over single gene analysis include noise and dimension reduction and may provide better biological interpretability [10]. Our assumption is supported by the recent large meta-analysis in African Americans with Alzheimer’s disease (AD), which found that while the major pathways involved in AD etiology are similar between non-Hispanic White and African American individuals, the disease-associated loci within these pathways differ [11]. Instead of a meta-analysis on the genotype data such as [11], we developed a pipeline, where we used a genetically regulated expression (GReX) imputation technique, computed differential expression probabilities on each population with regard to the phenotype, followed by the use of a computational systems biology method to identify gene modules enriched with the phenotype in question. Importantly, our statistical tests across populations were performed on the gene module level instead of on the gene level. We further used Gene Set Variation Analysis (GSVA) of the imputed expression [10] to evaluate our pipeline’s results.

We focused on AD to test our method, following the observations of [11] and used our method on three populations—Non-Hispanic Whites, African Americans and Hispanics to detect known and novel genes in modules and pathways associated with AD. In the following, we present our pipeline, followed by its evaluation on AD data.

## 2. Materials and Methods

### 2.1. Data

Genotype and phenotype data were downloaded from dbGaP. It includes data from three studies: (1) 2545 participants (1266 with AD and 1279 controls) from a multiplexed family study for late-onset Alzheimer’s disease including primarily self-reported Whites (European and North Americans), with 3% African American and 3% of Hispanic ancestry (LOAD CIDR Project, dbGaP Study Accession: phs000160.v1.p1), 1221 participants (85 with AD and 1136 controls) in a longitudinal, prospective population-based comparative epidemiological study of the prevalence and incidence rates of Alzheimer’s disease and other age-associated dementias in African Americans in Indianapolis and Yoruba living in Ibadan, Nigeria (dbGaP Study Accession: phs000378.v1.p1) and 3102 participants (1425 with AD and 1677 controls) in a late-onset Alzheimer’s disease study of Caribbean Hispanic populations (dbGaP Study Accession: phs000496.v1.p1). Imputation of the genotype files was conducted using the Michigan Imputation Server [12] and the Haplotype Reference Consortium (HRC) r1.1 [13].

### 2.2. Enriched Gene Pipeline

**Figure 1:**
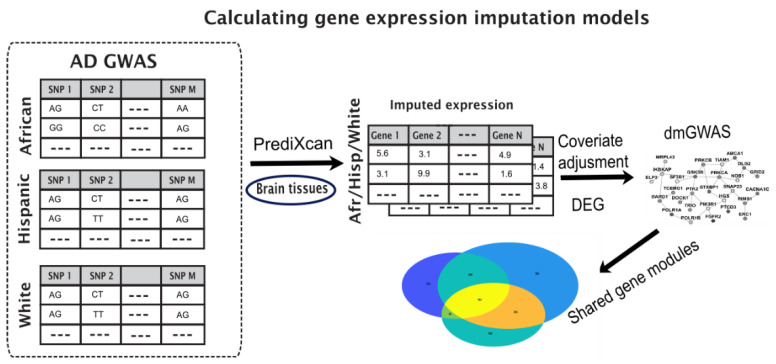
Illustration of the pipeline to identify shared gene modules between different populations. GWAS data is fed into PrediXcan for gene expression imputation, followed by adjustment for covariates and differential expression calculations. Genes with their *p*-values are fed into dmGWAS module to produce dense gene modules, which are compared to identify shared modules between populations.

We employed a pipeline to identify disease-associated gene modules from different populations. Our pipeline used differential expression tests on imputed gene expression from genotype data for each population and applied dense module searches on a protein interaction network (PPI). The steps of the pipeline are described below and illustrated in Figure 1.

### 2.3. Imputed Gene Expression Data

We applied the PrediXcan gene expression imputation tool (version 8 of Genome-Tissue Expression (GTEx) data) on each of the three AD genotypes studies independently. We imputed gene expression from 13 brain tissues available in GTEx that have been implicated in association with AD, including amygdala [14], anterior cingulate cortex ba24 [15], caudate basal ganglia [16], cerebellar hemisphere [17], cerebellum [18], cortex [19,20], frontal cortex ba9 [21], hippocampus [22], hypothalamus [23,24], putamen basal ganglia [16], spinal cord cervical c-1 [25]}, nucleus accumbens basal ganglia [16,26] and substantia nigra [27], obtaining imputed gene expression values per tissue ranging between 2040 and 6092 genes (3500 on average).

### 2.4. Identify Differentially Expressed Modules through Dense Gene Module Computations

We first adjusted the imputed expression for demographic covariates by retaining the residuals after fitting a linear model for the phenotype (AD cases vs. controls) using sex, age and four top principal components as covariates of the model (the number of principal components was selected based on the analysis of Chen et al. [28]), where the principal components serve to correct for potential population structure.

We performed a differential expression analysis on each adjusted imputed expression in each population and in each brain tissue using the limma algorithm [29] (a total of 39 differential expression results for three populations and thirteen tissues). For each population and in each brain tissue, the gene-specific *p*-values served as the discovery cohort for dense module searching and dual evaluation by dmGWAS [30,31] while the two remaining populations served as evaluation cohorts, in line with their previously published methodology. For example, we used the White population as the discovery cohort and the African American/Yoruban (Afr) and Hispanic (Hisp)as the evaluation cohort, the Afr as the discovery cohort and the other two as evaluation etc. We used the gene *p*-values to define the gene node weight as a Z-score transformation from φ^−1^ (1 − p), where p denotes the gene *p*-value from each population, and φ is the cumulative distribution function of the standard normal distribution function. Then, we performed greedy searching iteratively for each seed gene and expanded on the reference protein–protein interaction (PPI) network by combining the Z-score of each node. We used the default parameter setting to control the output module sizes. In short, discovered gene modules are defined as subgraphs within a protein interaction with the top combined module Z-scores. Each module was further permutated 1000 times to assess its significance compared with the null model. Lastly, we transferred the gene *p*-value to Z-score for the third population. And we combined the module scores for previous significant modules accordingly to assess their significance with the null model. The final output of the pipeline is sets of statistically significant gene modules associated with AD across populations.

Visualization of the shared genes on a PPI network was done using STRING [32] and Cytoscape [33]. The enrichment analysis of biological processes was done by Toppgene [34] via a hypergeometric test.

### 2.5. Gene Set Variation Analysis of the Imputed Expression

Gene set enrichment was performed for all the imputed AD samples using the R package “GSVA” (function gsva—arguments: method = “gsva”, mx.diff = TRUE) [10]. GSVA implements a non-parametric unsupervised method of gene set enrichment that allows an assessment of the relative enrichment of a selected pathway across the sample space. We collected three major sources of pathway and functional terms (canonical pathway, Gene Ontology, brain cell type-specific pathway, and brain cell type signature). Specifically, we obtained the curated KEGG, REACTOME, BIOCARTA pathways from Molecular Signatures Database (MSigDB 7.4 C2 category, access on 5 October 2021). We obtained the non-redundant Gene Ontology term from WebGestalt [35] (5 July 2021). Overall, we curated 2521 functional gene lists. The scores of each GSVA pathway from all samples followed approximately a bimodal distribution, representing their relative variation of signature pathway activity. Lastly, we used a limma package [29] to conduct a differential analysis between the mean of GSVA scores in AD samples and controls within each of three populations to identify significantly altered GReX at the pathway level.

## 3. Results

### 3.1. Key Gene Modules Are Shared across Populations

We employed a pipeline to identify disease-associated gene modules that bridge different populations based on imputed gene expressions from genotype data (Section 2, Figure 1). 

**Figure 2:**
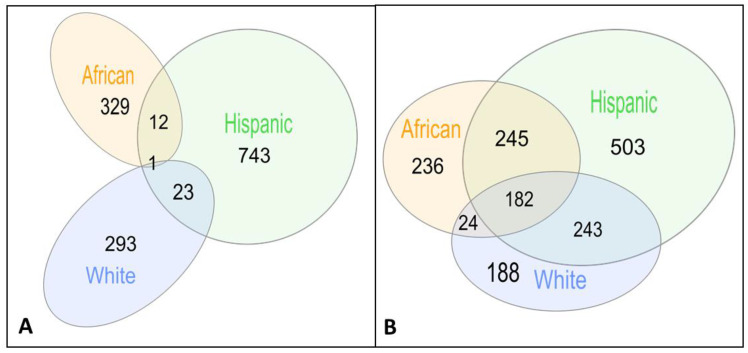
Venn diagrams of shared gene modules (**A**) and shared genes (**B**) between populations.

Briefly, we calculated gene probabilities for differential imputed expressions between AD cases and controls and fed these probabilities into a dense module searching s in protein–protein interaction networks (dmGWAS [30]), discovering 317, 342 and 779 gene modules on Whites, African American/Yoruban (Afr) and Hispanic (Hisp) populations, respectively (Table 1, Figure 2A, see Section 2). Notably, the largest number of gene modules was discovered in Hispanics, including population-specific modules and modules shared with the two other populations, resulting in a small number of shared gene modules shared between the African and White populations but not with the Hispanic population (24 modules, Figure 2A).

In order to apply our pipeline to AD, we used 13 brain tissues reported to be associated with AD (Methods). Twenty-four of the gene modules discovered in the Hisp population are shared with the White population and 13 with the Afr population (Table 2) [36,37,38,39,40,41,42,43,44,45,46]. 

### 3.2. Shared Genes Overlap with Previously Detected AD-Associated Genes

Some of the gene modules only partially overlap in their gene members between populations. We thus focused next on the shared genes within these gene modules, displaying larger overlap (Figure 2b). The significant gene modules include 637, 1173 and 687 genes in gene modules discovered in White, Hisp and Afr populations and validated in the complementary population, respectively (Figure 2b). A total of 182 of these genes appear in gene modules from all three populations (Table S1). Three genes of these 182 shared genes have recorded association with AD in Malacards (out of 209 genes) [47], DYRK1A [48,49], GPC1 [50,51,52] and PRNP [53,54,55]. Additionally, the POLR2E gene has been detected through another method that integrates GWAS, expression quantitative trait loci, and methylation quantitative trait loci data to identify AD-related genes [56].

### 3.3. Shared Genes Are Enriched with Biological Processes Associated with AD

We analyzed the known biological processes and diseases enriched with the 182 genes shared between all three populations. The top Gene Ontology biological processes enriched in this gene set are regulation of organelle organization (38 genes, FDR B&H *p* < 4 × 10^−7^). It has been previously reported that aberrant intracellular β-Amyloid (iAβ) interacts with several cell organelles, including accumulation commencing in endosomes, mitochondrial localization of iAβ and association of both key AD markers, iAβ and Tau, with endoplasmic reticulum and lysosome, serving as key organelles in the pathogenesis of AD [57,58,59]. Correspondingly, top enriched cellular components include the lumenal side of endoplasmic reticulum membranes (5 genes, FDR B&H *p* < 7 × 10^−4^) and the Golgi apparatus (34 genes, FDR B&H *p* < 8 × 10^−4^), where impaired Golgi morphology has a potential relationship to AD disease development [60]. 

Another significant biological process is regulation of cell cycles (33 shared genes, FDR B&H *p* < 2 × 10^−4^). The dysregulation of cell cycle control is suggested to be an integral part of AD [61]. Additionally, the top enriched pathway is FGF signaling pathway (10 genes, FDR B&H *p* < 2 × 10^−4^). Modulation of FGF receptor signaling is an intervention and potential therapy for myelin breakdown in AD [62,63]. Finally, the top two diseases enriched with our set are other neurodegenerative disorders, including Parkinson’s disease (40 genes, FDR B&H *p* < 2 × 10^−4^) and Amyotrophic lateral sclerosis (24 genes, FDR B&H *p* < 0.01).

**Figure 3:**
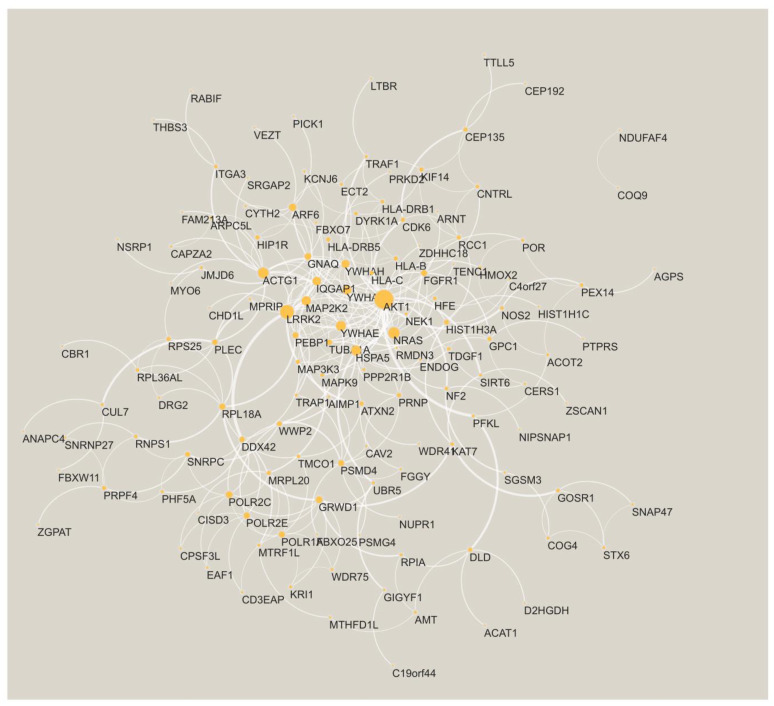
The PPI network is formed by the AD shared genes. The node size is proportional to its degree. The edge width is proportional to the confidence score from STRING [32].

Finally, we charted the PPI network spun by the shared genes. A total of 135 of the genes have at least one interaction with another gene (Figure 3). The two largest hubs in the PPI network are AKT serine/threonine kinase 1 (AKT1) and leucine-rich repeat kinase-2 (LRRK2). Both these genes are known to be associated with AD (see Section 4).

### 3.4. Comparison to GSVA

We compared the results obtained from dmGWAS to another well-established method to detect gene sets from GWAS. We conducted the gene set variation analysis (GSVA) among all AD cases and controls in Gene Ontology (GO) and canonical pathways (KEGG, REACTOME, BIOCARTA) (Table 2). We similarly used limma to conduct the differential GReX genes analysis in each population for AD cases and controls, adjusted for the same covariates as the dmGWAS (Section 2). Correcting for BH adjusted *p*-value < 0.05, only one REACTOME pathway survived in the White population, REACTOME_DISEASES_OF_GLYCOSYLATION (*q*-value < 0.008). We elaborate on this pathway’s relevance to AD in the discussion [50,51,52,64,65,66,67,68,69,70,71,72,73,74,75,76,77,78].

## 4. Discussion

In this work, we developed a pipeline to bridge genome wide association studies from different populations. We applied our pipeline to AD within three populations, African, Hispanic and White. While AD risk and severity vary significantly across racial/ethnic populations [79,80], previous publications showed that the major pathways involved in AD etiology in African American individuals were similar to those in non-Hispanic White individuals, despite the fact that disease-associated loci within these pathways differ [11], making AD an appropriate candidate for our methodology.

Our methodology is based on dense module searching for genome-wide association studies in protein–protein interaction networks [30], using differential expressions in imputed gene expression (GReX). In this work, we applied it in a way that iteratively uses each population for discovery and the other two populations for evaluation. We were able to identify shared gene modules that bridge the genetic information. We identified one gene module that was discovered and validated in all three populations and 182 genes that were shared across overlapping gene modules. These shared genes were indeed enriched for biological processes, pathways and neurodegenerative diseases associated with AD. Most of the previous genetic AD studies were performed on a single population, including studies using PrediXcan [28,80,81]. Notable attempts to bridge different populations include meta-analysis of genotype data from different populations such as African Americans and Whites [81] and transcriptional differences in AD between African American/African ancestry and Whites such as [82,83] that focused only on the APOE4 gene.

We further compared our methodology to GSVA, and applied it to the imputed gene expression. As opposed to the dmGWAS, this method only retrieved one pathway identified in only one population. This might suggest that the bridging of populations by the dmGWAS method is helped by incorporating protein interaction networks, thus considering also the connection between genes.

Only one module, including three genes C4orf27 (HPF1), KCNJ6 and TMEM218, is shared between the White and Afr populations and is also included in the 24 and 13 modules shared between Hisp, White and Afr populations, all identified in the brain cerebral hemisphere (another module that includes these three genes + IFNGR2 is shared between the Hisp and Afr populations). Interestingly, TMEM218 was identified as a common gene between obstructive sleep apnea and AD [36]. While KCNJ6 has no known direct association with AD, KNJC6 is associated with Down’s syndrome [37,38], which has a well-established increased risk for AD [39]. Similarly, C4orf27 has no reported direct connection to AD, but it is a critical modulator of the activity of PARP1 [40,41], which has known neurodegenerative activity in AD [42,43].

Another gene module partially shared between the three populations includes HLA-B and HLA-H, where the gene modules shared between Afr and Hisp populations include HEATR1 and UXS1 genes and PSMA4 and WDR11 in the gene module shared between Hisp and White (Table 2). Indeed, HLA-B*4402 affects both brain atrophy and cognitive decline [44] and is linked to potential AD risk and had interaction causing altered brain volume in non-Hispanic Caucasians [44], while HLA-B7, a HLA-B serotype, is associated with AD in Caucasian populations [45]. PSMA4 is also a gene bridging between mild cognitive impairment (MCI) and AD [46].

One REACTOME pathway survived in the White population, REACTOME_DISEASES_OF_GLYCOSYLATION (Table S3). Glycosylation has been identified as an early stage biomarker in AD [64], and is correlated with the severity of amyloid and tau pathology in both preclinical and clinical AD patients [65]; recent evidence implies that glycosylation of proteins and a number of other AD-related molecules is altered in AD, suggesting a potential implication of this process in disease pathology [66,67,68]. Out of this pathway, GPC1 (Glypican 1) appears in all the populations based on our dmGWAS pipeline (out of the 182 genes). As was mentioned, GPC1 already has substantial evidence for associating with AD [50,51,52]. 

Another two genes from this Reactome pathway are B3GAT3 (Beta-1,3-Glucuronyltransferase 3) and MPI (Mannose Phosphate Isomerase) that were discovered in the White and Hisp populations (but not the Afr population). B3GAT3 is one of the top genes with the lowest *p*-values significantly downregulated in 2 × Tg-AD mice [69] and defects in B3GAT3 in mice may affect the biosynthesis of chondroitin sulfate, dermatan sulfate and heparan sulfate [70], all directly associated with AD. Specifically, chondroitin sulfate is associated with the lesions of Alzheimer’s disease [70,71,72]; dermatan sulphate proteoglycan is substantially reduced in AD fibroblasts and presents in neurofibrillary tangles [73,74]; and a growing body of evidence from in vitro and in vivo studies suggests functional roles of heparan sulfate in Aβ pathogenesis in AD [75,76]. The second gene is MPI. MPI catalyzes the interconversion of mannose-6-phosphate whose overexpression increases Aβ secretion and alters expression profiles of genes involved in AD pathology [64,76,77].

Portraying the shared genes on a PPI network exposed highly connected genes (hubs) such as AKT1 and LRRK2. These genes are known to be associated with AD. Specifically, the PI3K-Akt Pathway (of which AKT1 is part) is part of altered insulin signaling in AD brains [84] and a target for the prevention and treatment of AD [85]; strong Akt immunoreactivity was seen in AD pyramidal neurons likely undergoing degeneration and in reactive astroglia [86]. Additionally, LRRK2 has variants associated with AD [87] and tau pathology is prevalent in individuals carrying a mutation previously associated with Parkinson’s disease [88]. 

Our methodology is not limited to AD. However, it is limited in the represented populations on which the GReX imputation model that we used was built and potentially biased by the imputation panel (HRC) that was used to impute the GWAS studies. Specifically, the majority of the samples in GTEx are from the European population, with a small portion of the African American population from the Genome-Tissue Expression (GTEx) dataset. GTEx v8 includes 84.6% of the samples identified as White, 12.9% as African American and 1.9% reporting Hispanic or Latino ethnicity. It thus might not fully reflect the GReX specificity in each population. Additionally, GTEx is limited in addressing other populations such as Asian populations. In addition to the GReX imputation models population bias, there is also population bias in the reference panels used for imputing the genotype arrays used in this study. The HRC panel biases non-European groups towards appearing more like Europeans. However, out of existing options, including the 1000 genomes and the Consortium on Asthma among African-Ancestry Populations in the Americas (CAAPA), HRC was reported as the better reference panel even for the imputation of genotyped African Americans [89]. Thus, more diverse gene-tissue measurements will be needed to improve our pipeline, and this is especially critical in AD.

Another limitation of our implementation is the imbalance between the sample size in each population. Specifically, the African population is the smallest and has the largest difference in case-to-control ratio. Additionally, the African population is characterized by greater levels of genetic diversity compared to non-African populations [90]. While our principal component analysis found only minor population stratification in any of the populations (explaining between 2.85% of the variance for the Afr population to 5.12% for the Hispanic population by the top ten principal components; see also Figures S1–S3 for projection of the samples on the top three principal components), this fact, together with the difference in sample size may have caused the African population to have the least amount of shared gene modules (Table 2). 

We identified associations between gene modules/genes and AD; further research will be needed to extract causal relationships from our findings.

## 5. Conclusions

Our work extends the dense module network-based approach (dmGWAS) to work with the gene expression imputation GReX. Our methodology can be applied to other complex conditions while our detected genes and gene modules provide new testable hypotheses for the etiology of AD. In particular, it can be applied to other neurodegenerative diseases in order to compare them to the modules discovered in AD.

## Figures and Tables

**Table 1:** Summary statistics for the studies used in our analysis.

	White	African	Hispanic
# of samples	2545	1221	3102
# of cases	1266	85	1425
# of controls	1279	1136	1677
Average age cases	72.9	80.8	79
Average age controls	NA	78.3	73
# of significant modules	317	342	779

**Table 2:** Shared modules between populations. Highlighted genes in bold are further discussed in the paper.

Afr + Hisp	Afr + White	Hisp + White
AGPAT4, CYB5A, DRG2, HSPA14	**C4orf27, KCNJ6, TMEM218**	ACP6, DHX36, SUCLG2
ANO5, GADD45GIP1, NUP50, RNGTT		AGPS, CBS, NIPSNAP1, TRAP1
ARPC5L, DYRK1A, S100A10, TTLL13		AGPS, CDK5RAP2, NIPSNAP1, NME7
BDKRB1, CERS1, HHATL		AGPS, MCUR1, NDUFAF4, NIPSNAP1
BTN2A2, HLA-DRB1, HLA-DRB5		AIMP1, CEP135, GCSH, SFI1
**C4orf27, IFNGR2, KCNJ6, TMEM218**		ALDH5A1, HSCB, MRPL35, NFXL1, VEZT
C4orf27, **KCNJ6, TMEM218**		ALDH5A1, HSCB, NFXL1, VEZT
CCDC146, HIP1R, VPS28		ATP6V0A1, CYTH2, SMPD3
DCDC2, FAM118A, NMU		**B3GAT3**, CSGALNACT2, GOSR1, GPR35, NRAS, SNAP47
ENAH, FYCO1, PRMT6, SMG7		BMP7, GNB2, GRB7, SERINC1, TDGF1
**HEATR1, HLA-B, HLA-H**		BTN2A1, HMGCR, TYW1
**HLA-B, HLA-H, UXS1**		**C4orf27, KCNJ6, TMEM218**
NDUFAF6, OTX1, RGS20		CAPZA2, HIP1R, KRI1, MYO6, RIN3
		CBL, RIPK1, TAB2, YWHAE
		COMMD2, COMMD4, TP53RK
		CPSF3L, GIGYF1, HSD17B14, SNRPC
		CSNK1E, CUL7, DDX42, MAPK9, RCC1
		CSNK1E, KIAA0101, LTBR, NBR1, TMEM259, TRIM4
		DERL1, RMDN3, SLC13A3, SRPR
		**GPC1**, ITGA3, RABIF, RPS25, ZDHHC18
		**HLA-B, HLA-H, PSMA4, WDR11**
		MRPL20, MTRF1L, NDUFAF4, PDPR
		NRAS, SLC4A7, SNAP47, UNC5B
		RIPK1, TAB2, TICAM1

## Data Availability

All relevant data used in this study are available through dbGaP and detailed in the data section of the manuscript. The synapse and other glia cells gene sets were downloaded from https://ctg.cncr.nl/software/genesets. Other datasets could be obtained via the resources described in the Methods.

## Supplementary Materials

The following supporting information can be downloaded at: https://www.mdpi.com/article/10.3390/cells11142219/s1, Figures S1–S3: Projections of the African, Hispanic and White populations on the top three principal components; Table S1: AD-associated genes from gene modules shared across populations. Table S2: Gene modules discovered in each population. Table S3: Gene Set Variation Analysis result of Benjamini-Hochberg adjusted *p*-values for 2521 Canonical pathways and Gene Ontology terms among three populations.
