# Supplementary material for: A Method for Bridging Population-Specific Genotypes to Detect Gene Modules Associated with Alzheimer’s Disease
Source: Cells. 2022 Jul 16;11(14):2219. doi: 10.3390/cells11142219 (PMC9319087; doi:10.3390/cells11142219)

Supplementary Figures

A method for bridging population-specific genotypes to detect gene modules associated with Alzheimer's disease

Yulin Dai[1], Peilin Jia[1] , Zhongming Zhao[1] and Assaf Gottlieb[1, *]

[1] Center for precision health, School of Biomedical informatics, University of Texas Health Science Center at Houston, Houston, TX, 77030

Figures

Figure S1. The projection of the African population samples on the top three principal components

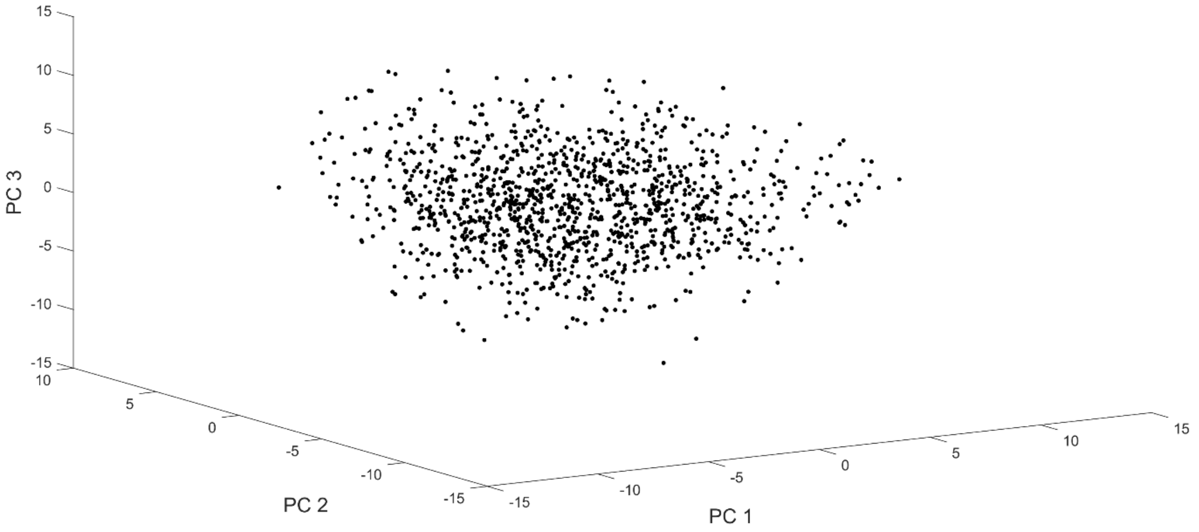

Figure S2. The projection of the Hispanic population samples on the top three principal components

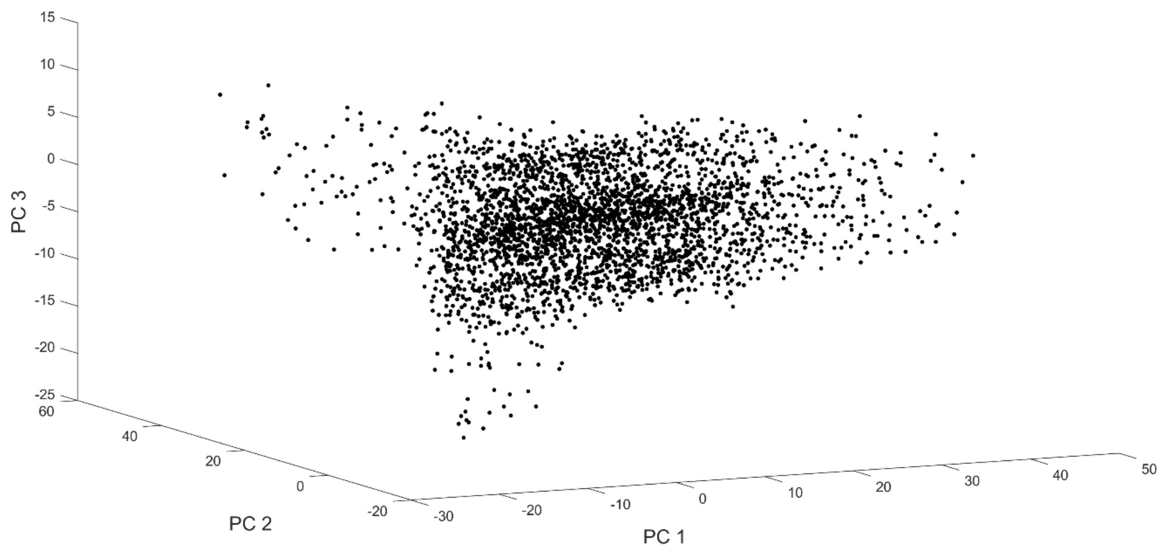

Figure S3. The projection of the White population samples on the top three principal components

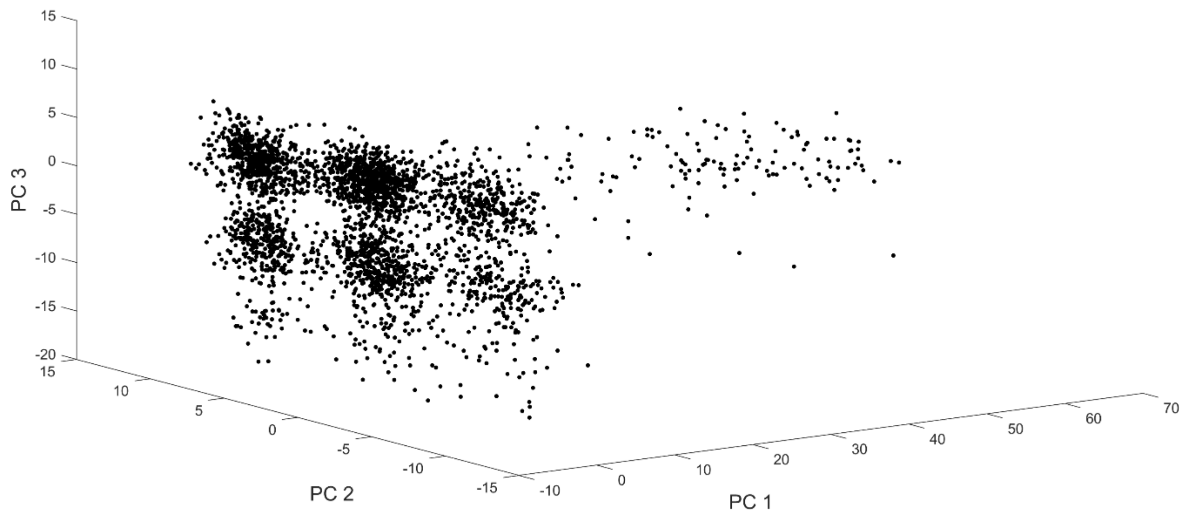

Supplement: Supplementary file 1 [file cells-11-02219-s001.zip › Supp_figures.pdf]
